# Factors not considered in the study of drug‐resistant epilepsy: Psychiatric comorbidities, age, and gender

**DOI:** 10.1002/epi4.12576

**Published:** 2022-01-07

**Authors:** Jesús Servando Medel‐Matus, Sandra Orozco‐Suárez, Ruby G. Escalante

**Affiliations:** ^1^ Department of Pediatrics Neurology Division David Geffen School of Medicine at University of California Los Angeles Los Angeles California USA; ^2^ Unit of Medical Research in Neurological Diseases Specialty Hospital “Dr. Bernardo Sepúlveda” National Medical Center S.XXI Mexico City Mexico

**Keywords:** hormones, psychiatric disorders, refractory epilepsy, sex

## Abstract

In basic research and clinical practice, the control of seizures has been the most important goal, but it should not be the only one. There are factors that remain poorly understood in the study of refractory epilepsy such as the age and gender of patients and the presence of psychiatric comorbidities. It is known that in patients with drug‐resistant epilepsy (DRE), the comorbidities contribute to the deterioration of the quality of life, increase the severity, and worsen the prognosis of epilepsy. Some studies have demonstrated that patients diagnosed with a co‐occurrence of epilepsy and psychiatric disorders are more likely to present refractory seizures and the probability of seizure remission after pharmacotherapy is reduced. The evidence of this association suggests the presence of shared pathogenic mechanisms that may include endocrine disorders, neuroinflammatory processes, disturbances of neurotransmitters, and mechanisms triggered by stress. Additionally, significant demographic, clinical, and electrographic differences have been observed between women and men with epilepsy. Epilepsy affects the female gender in a greater proportion, although there are no studies that report whether refractoriness affects more females. The reasons behind these sex differences are unclear; however, it is likely that sex hormones and sex brain differences related to chromosomal genes play an important role. On the other hand, it has been shown in industrialized countries that prevalence of DRE is higher in the elderly when compared to youngsters. Conversely, this phenomenon is not observed in developing regions, where more cases are found in children and young adults. The correct identification and management of these factors is crucial in order to improve the quality of life of the patients.

**Key Points**

- Most studies and clinical management of DRE generally do not consider factors such as age, gender, and the presence of comorbid disorders.
- In patients with DRE, neuropsychiatric comorbidities negatively impact the quality of life, increase severity, and aggravate prognosis.
- Age and gender of the patients are crucial factors in DRE but remain often under evaluated.
- Further research of impact and mechanisms of comorbidities, age, and sex in DRE is needed to design new effective and specific therapies.

## 1 INTRODUCTION

Epilepsy is a devastating condition that affects approximately 46 million people globally according to the 2016 Global Burden of Disease Collaborators.^1^, ^2^ Approximately one third of patients with epilepsy unsuccessfully respond to drug treatment; in other words, they suffer refractory epilepsy, also recognized as drug‐resistant epilepsy (DRE).^3^, ^4^, ^5^ The exact numbers of incidence and prevalence are not determined due to the diverse definitions of this condition and incorrect diagnosis. This multidimensional disorder involves several factors that directly affect the prognosis and effectiveness of the current treatments. A relevant aspect of most basic and/or clinical studies in the DRE research is that they usually do not contemplate factors such as age, gender, and the presence of comorbid disorders. For example, it has been shown that young and aged subjects are vulnerable populations to epileptic activity because of their physiological features.^6^ Particularly, elderly patients with DRE show a cognitive decline.^7^ On the other hand, regarding the effect of gender on epilepsy, pregnant women with epilepsy have increased complications including an increased risk of spontaneous miscarriage, antepartum or postpartum hemorrhage, hypertension, induction of labor, cesarean section, preterm birth, and fetal growth restriction.^8^ Additionally, patients with DRE frequently display additional neuropsychiatric disorders.^9^, ^10^ In some patients, it has been demonstrated that the impact of the comorbidities is even more severe than the epilepsy itself.^10^

The aim of the present review is to explore, describe, and analyze the importance of the existence of psychiatric comorbidities, the age and gender of the patients on the development of DRE, and the underlying mechanisms affected by these factors.

## 2 PSYCHIATRIC COMORBIDITIES IN PATIENTS WITH DRE

The concept of comorbidity is defined as the presence of one or more additional disorders that coexist with a primary condition, in this case, epilepsy.^11^, ^12^ It is known that in patients with epilepsy, particularly those that suffer DRE, the comorbidities contribute to worsening of the quality of life, increase the severity, and aggravate the prognosis of epilepsy.^10^, ^13^, ^14^ The study of this complex relationship is essential; however, it remains poorly understood.

Epidemiological studies have shown that the prevalence of psychiatric disorders is higher in people with epilepsy than in the general population.^15^, ^16^ For example, in the review published by Kwong and Park in 2014, the prevalence or frequency of anxiety and depression in patients with epilepsy was compared. They found that 9–36% of the patients were diagnosed with depression and 11–25% with anxiety. These results show that almost one‐third of them suffer from depression and anxiety; however, these conditions are often underrecognized and undertreated by clinicians.^17^ This is consistent with other studies that showed that mood disorders, followed by anxiety disorders (AD) are the most frequent psychiatric disorders in people with epilepsy.^16^, ^18^, ^19^, ^20^

The prevalence of epilepsy, as well as its neuropsychiatric comorbidities, in older adults is generally higher compared to younger ages.^6^ A study performed in 79 subjects, 50‐ to 67‐year‐old patients with DRE who underwent epilepsy surgery, was followed during 4.7 years (2–16 years). This follow‐up included EEG, MRI, and neuropsychological testing. Results showed that 58% of patients were seizure‐free. Neuropsychological impairment occurred in 13 patients in the form of depression and loss of concentration.^21^

Epilepsy may coexist with multiple psychiatric comorbidities.^22^ In an interesting study representing a good example of this multiple morbidity, a total of 52 patients with DRE underwent epilepsy surgery. Before surgery, one year and two years after surgery, all the subjects completed three different questionnaires in order to evaluate the presence and degree of depression, anxiety, and anger. Seizure outcome was excellent (81% were seizure free 1 year after surgery). Both anxiety and anger decreased significantly compared to the baseline, while depression showed a slow but non‐significant reduction. The authors attribute the discrete progression of depression to the fact that many people face difficulties in reorganizing their life even when seizures have disappeared.^23^

Another study evaluated psychopathology using a global assessment scale in 24 pediatric patients before and 2 years after epilepsy surgery. Psychiatric disorders (mainly attention‐deficit and hyperactivity disorder or autism spectrum disorders) were found in 70.8% of patients at some point. In 66% of the patients, there were improvements or no changes in psychosocial functioning 2 years after surgery. None of the seizure‐free subjects showed worst psychosocial functioning. The results proved the consequences of the extensive comorbidity in this cohort. Unfortunately, behavioral problems are usually identified only in 16% of the cases.^24^

Most of the available literature shows the association between DRE and its comorbidities with emphasis on the positive effect of the epilepsy surgery to reduce psychiatric disorders.^21^, ^23^, ^24^ However, in this case report the effect of the psychosis treatment on DRE was shown.^25^ Focal onset epilepsy with impaired awareness seizures accompanied by psychosensorial and psychotic symptoms is a rare condition.^26^, ^27^ There is no specific treatment for this particular type of epilepsy. The authors reported the case of a 21‐year‐old man diagnosed with schizophreniform disorder. The EEG showed an important subcortical epileptic activity with negative response to medication. Symptoms of this type of epilepsy were significantly improved using a psychotherapeutic treatment used for patients with psychotic disorders, known as integrated psychological therapy.^25^ Considering that antiseizure medications (ASMs) result ineffective, these findings suggest that psychotherapy may be a new treatment modality for patients with epilepsy with psychosensorial and psychotic symptoms.^25^

There is evidence suggesting an increased risk of suicide in patients with refractory epilepsy.^28^, ^29^ For example, the suicide risk and sleep quality in a cohort of 50 patients diagnosed with refractory epilepsy were evaluated.^30^ All patients had a detailed neurologic and psychiatric evaluation using validated questionnaires to identify suicide risk, sleep quality, emotional sensitivity, in terms of anger, fear, anxiety, and impulsivity. The results showed that suicidal patients have poor sleep quality compared with non‐suicidal subjects. Similarly, the patients with suicide risk showed an increased emotional sensitivity compared to the patients without suicide risk. These findings are relevant because sleep quality and psychiatric symptoms are rarely evaluated in patients with DRE.^30^

## 3 SHARED PATHOGENIC MECHANISMS BETWEEN DRE AND PSYCHIATRIC COMORBIDITIES

**FIGURE 1:**
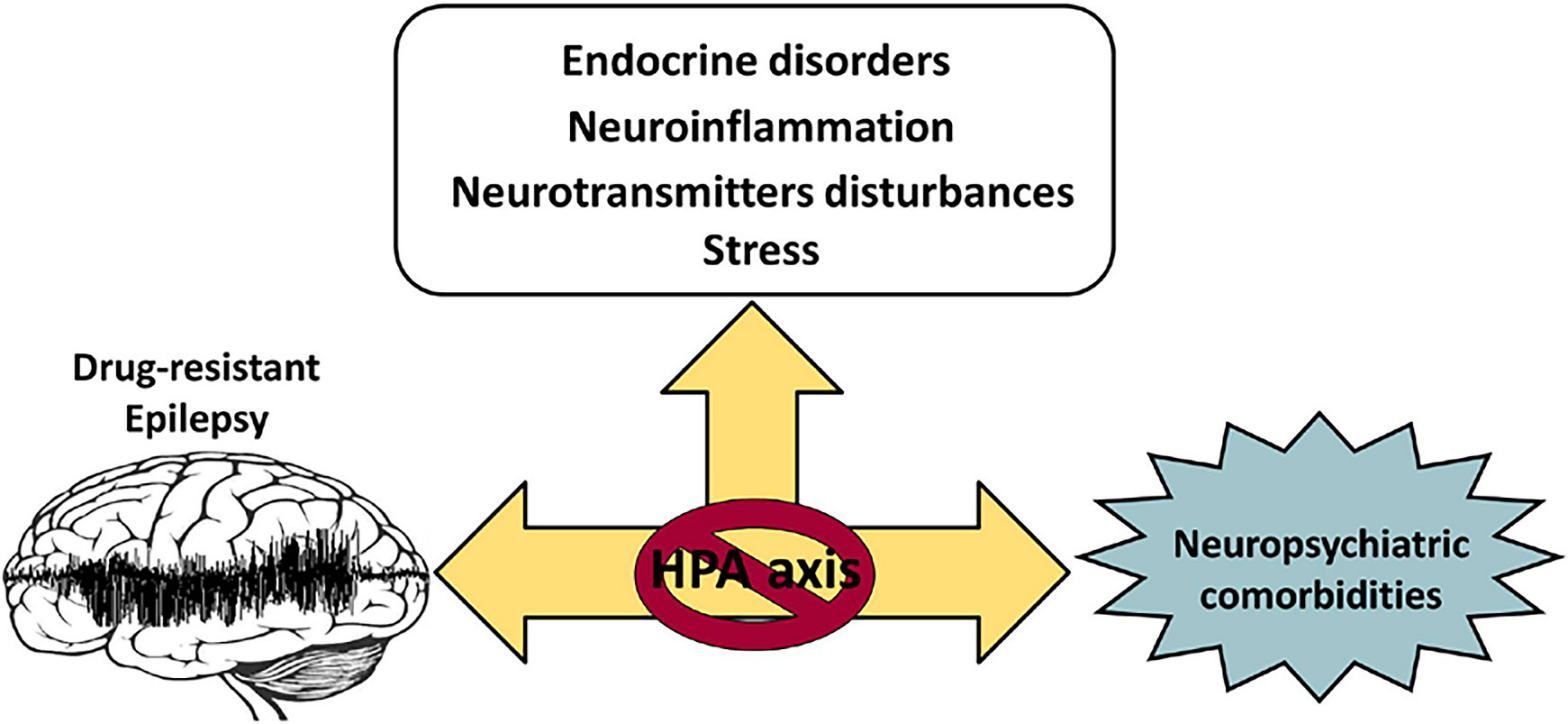
Potential shared mechanisms between psychiatric disorders and epilepsy. These processes occur as a result of a dysregulation of the hypothalamic‐pituitary‐adrenal (HPA) axis during epilepsy and, at the same time, they can be influenced by the presence of neuropsychiatric disorders. This suggests that the HPA axis participates in both epilepsy and its comorbidities

The close association between DRE and psychiatric disorders could be the result of the existence of common pathogenic mechanisms.^9^, ^31^ Previous studies in animal models have shown that some pathogenic mechanisms of mood and anxiety disorders facilitate the development of seizures.^9^, ^32^, ^33^, ^34^, ^35^ There is evidence in the literature of four different types of shared mechanisms between psychiatric disorders and epilepsy including endocrine disorders, neuroinflammatory processes, disturbances of neurotransmitters, and mechanisms activated by higher levels of stress (Figure 1).^31^

The endocrine disturbances produced by epilepsy may include a high serum concentration of cortisol as a result of a dysregulation of the hypothalamic‐pituitary‐adrenal (HPA) axis.^32^ Similarly, depression affects the HPA axis.^36^ In an interesting study, the effect of the administration of corticosterone in rats was analyzed. The treatment with corticosterone alone produced a significant reduction in the number of electrical stimulations necessary to reach a fully kindled status compared to the control and other glucocorticoid antagonists.^32^ In this context, the existence of a reduced expression of hippocampal glucocorticoid receptors in patients with drug‐resistant temporal lobe epilepsy and comorbid depression recently has been shown.^37^ Taken together, these findings suggest that the HPA axis is relevant in both DRE and depression.

The presence of cytokines in CNS during epilepsy also affects the HPA axis and participates in the modification of the expression of neurotransmitters,^38^ and neuropeptides.^39^ Some studies suggest that it is possible that the inflammatory response from microglia activation by cytokines can contribute to the development of psychiatric disorders. For example, Annamaria Vezzani and collaborators found that the administration of IL‐1beta in the hippocampus of rats increases the seizure duration.^33^

Glutamate is the major excitatory neurotransmitter in the cerebral cortex and plays an important role in mental disorders.^34^, ^40^, ^41^, ^42^ In another study, the level of glutamate in postmortem brains from patients with mental disorders was analyzed. The authors observed that the levels of glutamate in the brain tissue of major depression and bipolar disorder were significantly higher than control subjects. No changes were found in the brains of patients with schizophrenia.^34^

It has been shown that long‐term exposure to stressors can produce negative effects on mental health.^43^ The stress response is mediated by the HPA axis.^44^ As it was mentioned above, disorders such as depression, anxiety, and sleep disorders can influence the HPA axis.^45^, ^46^, ^47^ There are experimental data for the connection between stress and depression in the epileptic brain. For instance, in a rat model of comorbid depression and epilepsy, it has been detected the presence of an overactive HPA axis, with high serum corticosterone levels.^36^ In other investigations, the degrees of stress, depression, and anxiety between PWE, classified as well‐controlled epilepsy (WCE), poorly controlled epilepsy (PCE), uncontrolled epilepsy (UCE), and controls were evaluated. The levels of stress, depression, and anxiety were significantly higher in patients with UCE or DRE compared to all groups.^48^

## 4 INFLUENCE OF AGE AND GENDER ON EPILEPSY

Studies of incidence are especially useful in the epidemiology of epilepsy because they provide information on the etiology and prognosis of epilepsy. Thus, it is known that the mean estimated incidence of epilepsy is 43.4/100,000 among industrialized countries and 68.7/100,000 in developing countries, according to one of the first meta‐analyses.^49^ Prevalence data providing information on the burden of disease in a population show 2.7–7.1/1000 in developed countries and 2.2–22.2/1000 in low‐ and middle‐income economies.^50^ Data from a study in Rochester, Minnesota, one of the earliest and most comprehensive studies, show a cumulative incidence of 3.1% for epilepsy up to 74 years of age, and 4.1% for an unprovoked seizure of any type. Incidence by age shows a bimodal distribution in the industrialized countries, with an initial peak in childhood and a later peak after the age of 55 years. In the industrialized countries, the highest incidences are now seen in people over 75 years of age, reflecting improvements in care that have led to increased life expectancy.^51^ This incidence is not a feature in developing countries, where the highest rates of epilepsy are found in children and young adults, with relatively lower incidences in the elderly.^52^, ^53^ This indicates a major problem for the clinical setting, but also for public health. This is especially true as the population is living longer due to advances in medical treatments and greater knowledge about health and well‐being, particularly in developing countries.

On the other hand, it is noted that females have a slightly lower annual incidence of epilepsy than males, 46.2 versus 50.7/100 000, respectively, in the review by Kotsopoulos et al (2002). This gender difference may be multifactorial, but it is usually attributed to the greater exposure of males to risk factors for remote symptomatic epilepsy and acute symptomatic seizures, particularly head trauma, stroke, and CNS infections. Alcohol‐related seizures and alcohol‐related remote epilepsies are also significantly more common in men and account for much of this gender difference.^54^, ^55^

## 5 AGE AS A RISK FACTOR FOR THE DEVELOPMENT OF DRE

There are few studies by age and gender that associate a prevalence or incidence in DRE. Prevalence and incidence are useful for healthcare and resource planning, but are not consistently defined and tend to vary between studies, either due to problems with population selection, sample size, classification, or terminology. Prevalence and incidence studies should consider that different risk factors are related to drug resistance, such as seizure frequency, age of seizure onset, and number of ASMs. It has been documented in the literature that the treatment with multiple ASMs at an earlier age, in both patients and animal models of TLE, represent a more refractory phenotype, probably with difficulty to access diffuse epileptogenic networks formed by the intense epileptic activity and recently described as part of the DRE mechanisms^56^ that is less possible to be interrupted by surgery.^57^ This statement was supported by subsequent studies showing that daily seizures and early age of onset are risk factors for intractability in pediatric‐onset epilepsy. One of the earliest prospective studies showed that 13.9% of children with daily seizures developed DRE, compared with 6.8% of those with lower seizure frequency. In addition, the average age of seizure onset was lower in children with DRE.^58^ Another prospective study also found that daily seizures and age of onset were significant predictors of intractability,^59^ such that DRE affects the pediatric age group to a greater extent.

In addition, the cause or etiology of epilepsy is an important prognostic factor for recurrence; for example, focal epilepsies related to structural brain abnormalities are less likely to go into remission compared to those occurring in patients with normal brain imaging, even if they have a newly diagnosed epilepsy.^60^ In the same way, reviewing seizure patterns of children and adults with DRE showed that patients with TLE were significantly more likely to have DRE compared to all other patients with focal and generalized epilepsy, regardless of age.^61^ Interestingly, it has recently been demonstrated that anxiety disorder followed by mood disorders are the most common comorbidity in patients with refractory TLE.^62^ Similarly, several studies identifying early predictors of DRE in children^63^ and adults^64^ have shown that HS is highly correlated with drug resistance in pediatric age.^63^ However, a significant proportion [46%] of patients with TLE may have remission, suggesting, as in other populations, that some temporary cases have a more benign form.^64^, ^65^ In addition to etiology, the duration of epilepsy influences the severity of the disease. On average, in one cohort, patients with DRE had an evolution 6 years longer compared to controls without DRE. Similar findings were reported by a study of focal onset epilepsies in Korea [178 vs 102 months],^66^ and in a study conducted in Macedonia where patients with DRE suffered from the disease for 22 years and patients without seizures suffered from the disease for 14 years.^67^ In contrast, patients with a mild course of TLE are more likely to have late‐onset epilepsy and to have a shorter evolution. It should be noted that the longer the duration of epilepsy, the greater the number of ASMs tried. In general, a higher number of previously‐tested ASMs is associated with drug resistance.^67^ In addition to these factors, the DRE in the case of adults of increasing age with epilepsy involves having a history of years with metabolic or toxic factors (eg, drugs or excess alcohol) and depression. The increased prevalence of new‐onset seizures in persons aged ≥60 years is due to several factors associated with this special population (in addition to the increase in the aging population), including coexisting conditions (or comorbidities) such as cerebrovascular disease (eg, stroke), arterial hypertension, diabetes, and dementia.^6^ These comorbidities are common in persons aged ≥60 years, and for this reason, they are an important consideration when treating people with epilepsy.

## 6 GENDER AND AGE DIFFERENCES IN DRE

Steroid hormones influence brain development and seizure^68^ differences in endogenous steroid hormone levels and may promote variable susceptibility to seizures, as these hormones have been shown to affect the expression of Na‐K‐Cl cotransporters in a sex‐specific manner.^69^, ^70^, ^71^ It is likely that these changes contribute to variation in GABAergic inhibition. The immature brain has relatively higher concentrations of intracellular chloride ions,^72^ and the expression of two Na‐K‐Cl cotransporters (NKCC1 and KCC2) is responsible for this difference. In the neonatal brain, NKCC1 is more highly expressed and maintains high concentrations in neurons, and these proteins will eventually convert KCC2 cotransporters in neuronal membranes as the brain matures.^68^ Thus, chloride concentrations are determined in part by the ratios of NKCC1 and KCC2, as NKCC1 brings chloride ions into the cell and KCC2 extrudes them.^68^ When gamma‐aminobutyric acid (GABA) binds to its receptor in neonates, chloride ions leave the neuron at an increased rate, leading to neuronal depolarization. This explains why neonatal seizures are not sensitive to GABAergic drugs.^72^

Since it became known that during development, the brain‐derived neurotrophic factor (BDNF) regulates KCC2 expression in the same way, BDNF‐TrkB (TrkB as the receptor for the BDNF and NT‐4/5) modulates membrane insertion and function to KCC2,^73^, ^74^, ^75^ it has initiated the understanding of a novel role of KCC2 in the occurrence of refractory seizures in neonatal brains where KCC2 hypofunction has been documented.^76^ Rescue of KCC2 hypofunction by inactivation of the BDNF‐TrkB pathway^73^ or functional enhancement of KCC2 has been shown to rescue refractoriness in P7 CD‐1 mice. In neonatal seizures, it has been proposed that the ineffectiveness of the first‐line antiseizure targeting on the GABA_A_ receptor depends on the reversal of the Cl‐ gradient that is maintained by KCC2. Age‐dependent BDNF induction after seizure activity may underlie excessive BDNF release in the immature brain; for example, it was recently demonstrated that acute and transient antagonism of the TrkB receptor after stroke may be a novel and effective way to limit the onset of the drug‐resistant seizures in the neonatal brain.^74^ This transient blockade also rescued acute and subacute dysregulation of KCC2, which might otherwise be detrimental in a maturing brain, given the important role of KCC2 in dendritic spine formation, AMPA receptor trafficking, and the formation of functional GABA synapses.^75^

On the other hand, experimental evidence shows that the conversion of NKCC1 to KCC2 differs in a sex‐dependent manner.^70^ Females are known to undergo this conversion at an earlier developmental stage in certain sexually dimorphic brain regions, like the substantia nigra, hippocampus, and entorhinal cortex, although region‐specific differences have been reported. This sex‐specific difference in the timing of maturation of the GABAergic signaling has been proposed as an important contributing factor in the sexual differentiation of the brain, including cortical and subcortical networks involved in seizure control and response to GABAergic drugs, and this difference may underlie increased seizure susceptibility in male individuals.^69^, ^70^, ^71^, ^77^

The influence of cotransporters in refractory epilepsy in early developmental ages is known; however, in the case of older people, there are few studies. Most are focused on TLE, which is an epileptic syndrome associated with seizures that predominantly initiate in the temporal lobe and is often accompanied by temporal lobe pathology such as sclerosis, mossy fiber sprouting, and hippocampal degeneration.^78^, ^79^, ^80^, ^81^ The known causes of TLE are multiple, but often remain unknown.^82^ The ~40% of patients with TLE that become refractory usually end up opting for resective surgery to remove the seizure‐initiating focus.^83^ Subiculum tissue resected from refractory SLE patients with hippocampal sclerosis showed spontaneous interictal activity in vitro that correlated negatively with KCC2 expression.^84^ Similar studies have shown decreased KCC2 and increased NKCC1 transcription in resected TLE tissue.^85^ TLE has been widely associated with impaired Cl‐ homeostasis and depolarizing GABAergic transmission.^86^ TLE and hippocampal sclerosis have been associated with seizures in early life, especially febrile status epilepticus (FSE).^87^ Mutations in SLC12A5, the gene coding for KCC2, have been reported in patients with febrile seizures,^88^, ^89^ suggesting that mutations in their coding or regulatory regions affect Cl‐extrusion functions, which may contribute to genetic susceptibility to TLE.

Likewise, several mechanisms have been proposed to explain sex‐based differences in epilepsies and age‐independent seizure susceptibility^90^ but not in DRE. These factors include steroid hormones, cytochrome P450 activity, neurotransmitter systems, and gender‐associated neural networks in the brain.^90^, ^91^, ^92^ Among these factors, the impact of steroid hormones and endogenous neurosteroids on neuronal excitability and seizure susceptibility is one of the most investigated.^93^, ^94^, ^95^ Another factor is the neural networks that are different in relation to brain connectivity, as the male brain has better intrahemispheric neural communication due to a greater abundance of white matter connections between cortical regions.^96^, ^97^, ^98^ In contrast, the female brain exhibits greater interhemispheric connectivity and local clustering.^96^, ^97^, ^98^ This discrepancy has been described in both adolescent and adult brains and may partly explain why more idiopathic generalized epilepsies are diagnosed in females than in males.^96^, ^97^, ^99^ The same occurs in the limbic system and may be a determining factor in the establishment of TLE.^96^, ^97^ TLE is one of the most common forms of epilepsy and is characterized by spontaneous focal seizures originating in the limbic system. Within the amygdala, males have been shown to have better connectivity on the right side, whereas females have more neural connections on the left side.^100^, ^101^ Taken together, these structural variations within the limbic system suggest that sex differences in epilepsies may be explained in part by underlying sex‐based variations in functional connectivity. On the other hand, steroid hormones are synthesized and secreted from ovarian, gonadal, and adrenal sources, and play a key role in the neuroendocrine control of neuronal excitability and seizure susceptibility.^93^

Progesterone is an antiseizure hormone that has a relatively higher concentration in women; its antiseizure effect is associated with the modulation of glutamatergic (excitatory) neurotransmission.^102^ On the other hand, estrogens may affect seizure susceptibility because they have proseizure and epileptogenic properties in animals and humans.^103^ The effect of estrogens on hippocampal seizure susceptibility is controversial.^103^ Although estradiol has been shown to be proseizure in several studies, there is also evidence to support the lack of effect or protective effect of estrogens.^104^, ^105^, ^106^ Basic studies have shown that estrogens positively impact glutamatergic transmission and produce proseizure actions in the brain.^107^ However, other studies suggest that estrogen may also exhibit antiseizure properties primarily through the neuroprotective effects of the β‐estradiol subtype, showing that the impact of progesterone on seizure susceptibility in women is much more profound.^106^, ^108^

**FIGURE 2:**
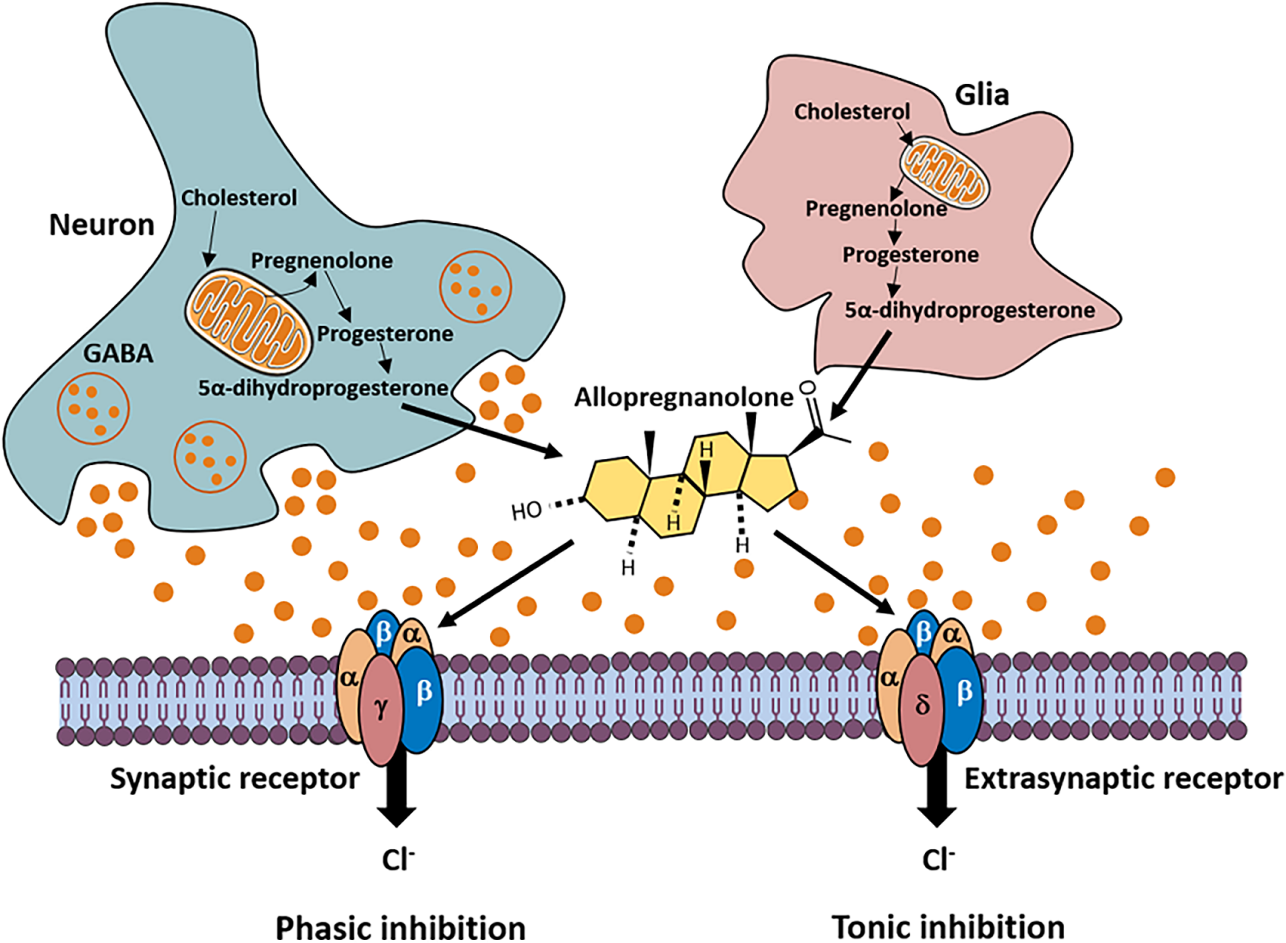
Neurosteroids as local modulators of inhibitory neurotransmission. GABA released from vesicles activates a family of postsynaptic GABA_A_ receptors resulting in a momentary inhibitory postsynaptic current known as phasic response. Neurosteroids released locally from neurons or glia augment these responses. Furthermore, GABA can also produce a tonic inhibition by acting on extrasynaptic GABA_A_ receptors, and this conductance can have a considerable impact on neuronal excitability

The influence of neurosteroids on both neuronal excitability and inhibition has been associated with changes in GABA_A_ receptor (GABA_A_R) expression. In addition, fluctuations in progesterone and progesterone‐derived neurosteroids during the menstrual cycle have been proposed to alter GABA_A_R‐mediated tonic inhibition.^109^, ^110^, ^111^ In another study, extrasynaptic δ‐containing GABA_A_R were shown to be crucial mediators of changes in neuronal excitability and seizure susceptibility in mice.^112^ On the other hand, changes related to the estrous cycle produce changes in the expression of δ‐containing GABA_A_R in different regions of the hippocampus consequently in tonic inhibition^113^ (Figure 2), as well as seizure susceptibility. These findings are highly relevant to the low incidence or severity of seizures in females. Sex differences in neurosteroid protection are not related to pharmacokinetic factors; nevertheless, with a higher abundance of δ‐containing GABA_A_R in females, neurosteroids produced a greater potentiation of tonic currents in female hippocampal neurons than in males. Thus, it is likely that neurosteroids protect against seizures in females because of the high abundance of the extrasynaptic δ‐containing GABA_A_R in the hippocampus and other brain regions.^114^ However, it is likely that other factors, such as differences in phosphorylation and trafficking in phosphorylation, contribute to sex differences in the antiseizure effectiveness of neurosteroids through GABA_A_ receptor.

The studies noted above have shown that neurosteroids are GABA_A_ receptor modulators, which are synthesized from progesterone and mediate protective effects in the brain.^115^ However, gender differences in the antiseizure activity of progesterone have not been extensively studied. Studies in animal models tested the ability of progesterone and neurosteroids to protect against pentylenetetrazole (PTZ)‐induced seizures in male and female mice.^116^ Progesterone protected both male and female mice against PTZ‐induced seizures in a dose‐dependent manner. However, in females, progesterone antiseizure activity was higher in a dose‐response mode. Since the antiseizure activity of progesterone is mainly due to its metabolic conversion to the neurosteroid allopregnanolone,^116^ the protective activity of allopregnanolone in male and female mice in PTZ‐induced seizures was evaluated. Like progesterone, allopregnanolone protected mice in a dose‐dependent manner, and the dose‐response curve of the neurosteroid was higher in females than in males. Using progesterone receptor (PR) knockout mice, it was confirmed that gender‐related differences in the antiseizure potency of progesterone or allopregnanolone are not due to differences in PRs in the brain.^116^ These results agree with previous studies, suggesting that females are more sensitive to the actions of progesterone in the CNS than males.^117^ The gender‐related difference in progesterone protection is not due to known differences in the density or distribution of progesterone receptors in female and male brains.^118^, ^119^ However, the role of sexual dimorphism or the brain region involved remains unclear. It is possible, however, that endocrine differences between females and males are responsible. It has been established that testosterone and related androgens do not have antiseizure effects.^120^, ^121^ Alternative explanations include gender differences in the bioavailability or pharmacokinetics of progesterone and allopregnanolone or differences in the sensitivity of GABA_A_ receptors to these compounds.

In addition, testosterone has a marked impact on seizure susceptibility through its metabolism to estrogen.^122^ Thus, it is important to lay the groundwork for understanding how sexually dimorphic GABAergic tonic circuits in the hippocampus may contribute to sex differences in seizure susceptibility and neurosteroid protection. These findings could be especially important for the design of personalized neurosteroid therapies for epileptic seizures, such as focal onset impaired awareness seizures, catamenial epilepsy, and status epilepticus.^91^

## 7 DISCUSSION

The treatment of epilepsy represents a challenge due to the complexity and diversity of the mechanisms associated. While in the preceding years notable advances in the neuroscience field have occurred, such as improvements in diagnostic techniques and more pharmacological therapies, the epidemiological investigations indicate that these improvements are not reflected in broad benefits for pharmacoresistant patients.^123^ This is more evident in DRE since the accessibility to pharmacological agents to stop or at least reduce the epileptic seizures is not yet a feasible option. Frequently, the patients with DRE have to deal with multiple adversities on their search for a solution for their devastating condition. For example, approximately 5% of the patients with DRE enter in a seizure remission period that can last for years, which provides these patients with a false hope. However, a considerable proportion of them (>70%) relapse after the 12 months following a remission.^124^

As it was mentioned earlier, psychiatric comorbidities have been associated with significantly decreased quality of life, increased disability, increased medication, and medical costs in persons with DRE. Furthermore, it has been revealed that the age of patients is also an important factor that determines the type of comorbidity related to epilepsy. In children with epilepsy, the most prevalent comorbidities include attention‐deficit hyperactivity disorder, mood and anxiety disorders, and autism spectrum disorder.^125^ On the other hand, disorders such as depression and anxiety disorder are frequently observed in adults.^126^ These neuropsychiatric comorbidities in patients with DRE are yet highly underdiagnosed and undertreated and must receive particular attention during the evaluation of this disease. The correct identification and treatment of comorbidities in DRE is critical. It has been shown that approximately two‐thirds of premature deaths in patients with epilepsy are attributed to comorbid diseases.^127^

A deeper examination of each patient with DRE must be carried out by physicians and neurologists in order to identify particular factors that may be related to either the joint development of neuropsychiatric comorbidities and/or the ineffective response to medication. This exploration could result in a better understanding of every case and consequently in a possible solution. There is clear evidence that shows that patients with a family psychiatric history are more likely to develop pathologic reactions to stressors.^128^ Particularly, family psychiatric history plays an essential role in pharmacoresistance^128^, ^129^; unfortunately, it is frequently under evaluated.

Aside from the potential mechanisms described in this review to explain the relationship between DRE and psychiatric comorbidities, some studies have suggested that this connection is due to the localization of the epileptic foci; however, the findings obtained so far are contradictory.^130^, ^131^, ^132^ For example, a study performed in 540 adult patients assessed the presence of anxiety and its association with the epilepsy type. The results showed an independent association of anxiety symptoms with focal epilepsy versus generalized epilepsy.^133^ Conversely, in another study, 144 patients evaluated for epilepsy surgery received psychiatric examination and it was found that psychotic syndromes were linked to a history of febrile convulsions and left‐sided temporomesial epileptogenic foci.^134^ Some investigations are pioneers in the study of this intricate subject. For instance, the existence of a relationship between a dysfunction in the self‐identity in patients with chronic focal epilepsy has been shown.^135^ The authors found a poor self‐identity development in patients with seizures arising from a focus in the mesial temporal compared to non‐mesial temporal seizure foci. This effect might be modulated by the extension of the seizure foci and the timing of seizure onset.^135^ Furthermore, the same group of investigators found that in patients with epilepsy with mesial temporal foci, objective verbal memory dysfunction, neuroticism, and female gender predicted memory complaints.^136^ Another study revealed that patients with temporal epilepsy had a higher prevalence of psychiatric comorbidities, predominantly anxiety, than patients with extra‐temporal epilepsy.^62^ Nevertheless, further studies are necessary to identify which disorders of the wide variety of psychiatric comorbidities of DRE can be linked with each type and/or localization of epilepsy.

Regarding the relevance of age on epilepsy, as was described above, it has been revealed that both epilepsy and its neuropsychiatric comorbidities are most frequent in elderly people.^21^ A limited number of studies have compared characteristics of epilepsy in older adults versus younger individuals,^137^ and even less is known about this matter in DRE. Certainly, the aged population is rapidly growing mainly in developed countries; therefore, more studies focused on the mechanistic processes of DRE, alone or in combination with other neurological disorders, on each stage of life are needed with the purpose of generating specific treatments and or preventive actions.

Over several years, most of the experimental models used in the study of epilepsy have been performed predominantly in males. Nevertheless, the effect of sex hormones on this condition has been well‐known.^138^ Some investigations have included both sexes in the study of DRE.^139^, ^140^ However, at the present time and further, it is essential to take this important factor into account in all experiments in order to find the specific differences in the development and management of DRE in each gender with subsequently extrapolation of the obtained findings for the benefit of the patients with DRE. Although studies have not been done in DRE, neurosteroids hold great promise for the treatment of epilepsy. A better understanding of the mechanisms of sex differences in the actions of neurosteroids may offer improvements in treatment strategies for sex‐specific forms of epilepsy.

Novel approaches in the research of DRE represent areas of opportunity for the improvement of its management. For example, presently there is scarce evidence regarding the existence of a difference in the gut microbial composition between patients with DRE and controls,^100^ and there is only one study, until now, that reported a patient with epilepsy who became seizure‐free after fecal microbiota transplantation.^141^, ^142^ Furthermore, limited exploration has been conducted to understand the relationship between gut microbiome and DRE. Some potential mechanisms include modifications of probiotics, prebiotics, or antibiotics. Future research may establish whether altering bacterial composition of the gut microbiome may help in the treatment of DRE.

Finally, it is important to note that new treatment approaches have been proposed in the recent years to address the resistance to current ASMs with promising results. For example, phytoconstituents, essential oils, endocannabinoids, and phytocannabinoids have shown antiseizure properties by a modulation of GABAergic neurotransmission.^143^, ^144^, ^145^, ^146^ Further studies are required to validate the effectiveness of these new alternatives in the treatment of DRE as a multifaceted condition.

## 8 CONCLUSIONS

In conclusion, the diagnosis and management of the psychiatric comorbidities in patients with refractory epilepsy represents a critical factor in order to reduce their impact on the development of this condition and improve their response to pharmacological treatment. Furthermore, the identification of these comorbidities could be a predictor of poor quality of life and may help to avoid severe consequences such as suicide risk. Unfortunately, at the present time, the investigation of psychiatric disorders by the physicians who treat epilepsy is not routine in most cases.

On the other hand, a deeper understanding of the molecular and neural network basis of age and sex differences in seizure and drug response is needed to design effective sex‐ and age‐specific therapies for epilepsy and identify whether these differences are also associated with DRE, and whether hormones can be reliable alternatives in the treatment of this complex neurological disorder.

## CONFLICTS OF INTEREST

The authors declare no conflicts of interest.

## ETHICAL APPROVAL

We confirm that we have read the Journal's position on issues involved in ethical publication and affirm that this report is consistent with those guidelines.
